# Clinical Utility of Rapid Pathogen Identification for Detecting the Causative Organisms in Sepsis: A Single-Center Study in Korea

**DOI:** 10.1155/2018/1698241

**Published:** 2018-08-27

**Authors:** Won-Young Kim, Eun Suk Jeong, Insu Kim, Kwangha Lee

**Affiliations:** Division of Pulmonary, Allergy and Critical Care Medicine, Department of Internal Medicine, Pusan National University School of Medicine, Busan 49241, Republic of Korea

## Abstract

**Purpose:**

The aim of this pre- and postintervention cohort study was evaluating how effectively rapid pathogen identification with matrix-assisted laser desorption/ionization time-of-flight mass spectrometry (MALDI-TOF MS) detected the causative organisms in sepsis.

**Methods:**

All consecutive adult patients who had bacteremia within 72 h of intensive care unit admission and met ≥2 quick Sequential Organ Failure Assessment criteria at intensive care unit admission were analyzed. The patients whose microorganisms were identified via MALDI-TOF MS between March 2014 and February 2016 formed the postintervention group. The patients whose microorganisms were identified by using conventional methods between March 2011 and February 2013 formed the preintervention group.

**Results:**

The postintervention group (*n*=58) had a shorter mean time from blood draw to receiving the antimicrobial susceptibility results than the preintervention group (*n*=40) (90.2 ± 32.1 vs. 108.7 ± 43.1 h; *p*=0.02). The postintervention group was also more likely to have received active antimicrobial therapy by the time the susceptibility report became available (77% vs. 47%; *p*=0.005). Its 28-day mortality was also lower (40% vs. 70%; *p*=0.003). Univariate analysis showed that identification via MALDI-TOF MS (odds ratio, 0.28; 95% confidence interval, 0.12–0.66; *p*=0.004) and active therapy (odds ratio, 0.38; 95% confidence interval, 0.16–0.95; *p*=0.04) were associated with lower 28-day mortality.

**Conclusion:**

Rapid microorganism identification via MALDI-TOF MS followed by appropriate antimicrobial therapy may improve the clinical outcomes of patients with sepsis.

## 1. Introduction

Sepsis is a life-threatening organ dysfunction caused by excessive responses to infectious pathogens by inflammatory mediators. Severe sepsis and septic shock are major healthcare problems that affect millions of patients globally each year [1]. Septic shock is the most common cause of death in intensive care units (ICUs), with mortality rates as high as 40–60% [2]. Current sepsis guidelines recommend that the administration of antimicrobials should be initiated as soon as possible [3] because any delay in effective antimicrobial therapy may decrease survival [4].

Blood culture is considered to be the key method for diagnosing the microorganisms that cause sepsis [5]. However, it is a time-consuming process and therefore often fails to provide the time-critical results needed for optimal early management. Moreover, the long delays associated with culture methods oblige physicians to begin empirical treatment with broad-spectrum antimicrobials, even though this therapy may not be optimal for the specific infection [6]. Moreover, excessive exposure to broad-spectrum antimicrobials promotes the risk that isolates develop antibiotic resistance [7]. Therefore, to reduce the use of empirical antibiotics, novel and more efficient diagnostic tools are needed.

Matrix-assisted laser desorption/ionization time-of-flight mass spectrometry (MALDI-TOF MS) is a relatively new technology for the identification of pathogenic organisms. It accurately and in particular promptly identifies most bacterial and yeast species [8, 9]; compared to conventional methods, implementation of MALDI-TOF MS decreases the time to organism identification by more than 1 day [10, 11]. Furthermore, MALDI-TOF MS testing of specimens taken directly from positive blood cultures has been reported to decrease the time needed to obtain antimicrobial susceptibility results [12]. Thus, it is conceivable that MALDI-TOF MS could serve as a complementary method in the critical care setting, such as in cases of sepsis or septic shock. Indeed, several studies have also reported that the introduction of MALDI-TOF MS reduces the ICU and hospital stays of patients with bacteremia and/or candidemia and markedly decreases hospital costs [10, 11, 13–15]. To our knowledge, however, the impact of MALDI-TOF MS on the clinical outcomes of patients with sepsis, especially those who are so ill that they must be admitted to the ICU, remains poorly understood. To address this, this study evaluated the effectiveness with which MALDI-TOF MS detected the causative organisms in patients with sepsis whose condition was severe enough to warrant admission to the ICU.

## 2. Materials and Methods

### 2.1. Study Design and Study Subjects

In this pre- and postintervention quasi-experimental study, we reviewed the medical records of all consecutive patients with sepsis who were admitted between March 2011 and February 2016 to the ICU of a 1,100-bed university-affiliated tertiary care hospital in Busan, Korea. Our hospital consists of six functionally separate ICUs with 85 beds: the medical ICU (12 beds), the surgical ICU (10 beds), the cardiac/stroke unit (14 beds), the neurosurgical ICU (13 beds), the emergency department ICU (20 beds), and the trauma ICU (16 beds). Each ICU has full cardiovascular facilities, close airway monitoring, and at least one full-time intensivist. All patients were managed by adherence to therapeutic recommendations based on the Surviving Sepsis Campaign Guidelines and the lung-protective ventilation strategy [3, 16]. Patients were included in the study if they were adults (aged ≥ 18 years), (i) had a documented bloodstream infection within 72 h of ICU admission and (ii) met ≥2 quick Sequential Organ Failure Assessment (qSOFA) criteria [17] at the time of ICU admission. Patients were excluded if they were not admitted to the ICU, were transferred from an outside hospital with an active bacteremia, the organisms had not been identified by MALDI-TOF MS at the time of this study, the patient died before the index blood culture became positive and/or treatment was withdrawn. For patients with multiple episodes of bacteremia, only the first episode was included.

The patients were divided into pre- and postintervention groups according to whether their ICU admission was before or after MALDI-TOF MS was introduced into the hospital (March 2013–February 2014). Thus, the preintervention group consisted of all septic patients who were admitted in the 2 years before MALDI-TOF MS was introduced, namely, between March 2011 and February 2013. Their sepsis-inducing microorganisms were identified by using conventional methods. The postintervention group consisted of all septic patients who were admitted in the 2 years after MALDI-TOF MS was introduced, namely, between March 2014 and February 2016. Thus, their sepsis-inducing microorganisms were identified by using MALDI-TOF MS.

The primary study outcome was time to receipt of the antimicrobial susceptibility results. Secondary outcomes included the frequency of active antimicrobial therapy by the time the susceptibility report became available; 28-day mortality after ICU admission; duration of mechanical ventilation (MV); and ICU and hospital lengths of stay. The study protocol was approved by the Institutional Review Board of Pusan National University Hospital (C-1703-004-052), which waived the requirement for informed consent because the study was retrospective. The study was conducted according to the tenets of the 1964 Declaration of Helsinki and its amendments. The patient records were anonymized and deidentified prior to analysis.

### 2.2. Data Collection and Definitions

Baseline demographic and clinical data included age, sex, comorbidities, source of bacteremia, dates of hospital and ICU admission, and date of initiation of MV (if applicable). The severity of illness at the time sepsis was diagnosed was assessed by measuring the severe inflammatory response syndrome (SIRS) criteria and the qSOFA score at the time of ICU admission according to the guidelines [17, 18]. The status of the patient within 72 h of ICU admission, namely, whether the patient was being treated with MV, neuromuscular blockers, vasopressors, and/or renal replacement therapy, was also assessed. Microbiological information included Gram-stain status, microorganism identification, and antimicrobial susceptibility test results. In addition, the antimicrobials used from the time of blood draw until the susceptibility report was received were extracted. This therapy was defined as active if it included one or more antimicrobial agents to which the causative pathogen was susceptible *in vitro*. It was defined as inactive if the blood isolate was resistant to the agents that were used (or no antimicrobial agents were used).

### 2.3. Microbiology Workflow

Blood culture samples were sent to the microbiology laboratory, and culture specimens were inoculated on appropriate solid agar media as soon as they were received. At the same time, all specimens were Gram-stained. For all positive blood cultures, organism identification was performed via one of the two methods. In the preintervention group, the microorganisms that grew were identified by conventional and automated biochemical methods (VITEK-2; bioMérieux, Marcy l'Etoile, France). In the postintervention group, identification was performed by MALDI-TOF MS (Bruker Daltonics, Bremen, Germany) running the Biotyper software version 3.0. In both the groups, antimicrobial susceptibility testing was performed by using the VITEK-2 and E-test (bioMérieux, Marcy l'Etoile, France) according to the same procedure. Identification and antimicrobial susceptibility testing were performed once per day at 9:00 AM in both groups. Thus, the only significant difference in the overall microbiology workflow between the groups was the method of microorganism identification.

### 2.4. Statistical Analysis

Continuous variables are presented as mean ± standard deviation. Categorical variables are presented as percentages. The two groups were compared in terms of continuous variables by using the Student's *t*-test and in terms of categorical variables by using chi-squared or Fisher's exact tests. Binary logistic regression was used to identify factors predicting 28-day mortality. Kaplan–Meier curves were generated to compare the 28-day survival of the groups. Kaplan–Meier survival estimates were also stratified by both pre/postintervention and active/inactive therapy to determine the ability of the intervention with initial active (or inactive) therapy to predict mortality. All tests of significance were two-tailed. *p* values < 0.05 were considered statistically significant. All analyses were performed by using SPSS version 18.0 for Windows (SPSS Inc., Chicago, IL, USA).

## 3. Results

**Figure 1:**
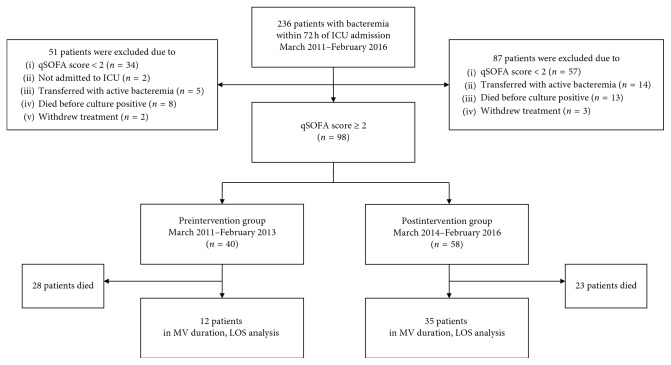
Study flow diagram. ICU: intensive care unit; qSOFA: quick Sequential Organ Failure Assessment; MV: mechanical ventilation; LOS: length of stay.

During the two study periods, 236 patients with bacteremia were admitted to the ICU. Of these, 91 (39%) and 145 (61%) were in the pre and postintervention groups, respectively. Of the 91 patients in the preintervention group, 51 were excluded because their qSOFA score at ICU admission was <2, they were not admitted to the ICU, they were transferred from an outside hospital with an active bacteremia, the patient died before the index blood culture became positive, or treatment was withdrawn. Of the 145 patients in the postintervention group, 87 were excluded because their qSOFA score at ICU admission was <2, they were transferred from an outside hospital with an active bacteremia, the patient died before the index blood culture became positive, or treatment was withdrawn. Thus, after exclusion criteria were applied, 98 patients with a qSOFA score of ≥2 at ICU admission were included in the final analysis. Of these, 40 patients (41%) were in the preintervention group and 58 patients (59%) were in the postintervention group (Figure 1).

The baseline characteristics of the two groups are shown in Table 1. The groups did not differ in terms of age, sex, or comorbidities, with the exception that malignancies were more frequently observed in patients in the preintervention group (45% vs. 16%; *p*=0.001). The groups did not differ in the mean number of SIRS criteria that were met or the mean qSOFA score at ICU admission (*p*=0.59 and *p*=0.68, resp.). The groups were also similar in terms of the need for MV, neuromuscular blockers, or vasopressors. However, the preintervention group tended to be more likely to require renal replacement therapy within 72 h of ICU admission (50% vs. 31%; *p*=0.06). In both the groups, the main origin of the bacteremia was respiratory, which accounted for 43% of the bacteremia cases in both groups. The next most common source was intra-abdominal infection (Table 1).

Regarding the 91 patients who were excluded from the study because they had a qSOFA score of <2 at ICU admission, the main origin of bacteremia was also respiratory (27/91, 30%), followed by intra-abdominal infection (22/91, 24%). The included and excluded patients did not differ in terms of bacteremia source, although the excluded patients did tend to have fewer patients with pneumonia (43% vs. 30%; *p*=0.06) and more patients with musculoskeletal infection (13% vs. 23%; *p*=0.08).

Table 2 shows the microorganisms that were responsible for the bacteremia in the study patients. The pre- and postintervention groups did not differ significantly with regard to the prevalence of Gram-positive, Gram-negative, yeast, polymicrobial, and multidrug-resistant organism bacteremia.

All antimicrobials used between the time of blood draw and receipt of the susceptibility report are shown in Table 3. The pre- and postintervention groups generally did not differ in terms of antimicrobial regimen. However, the preintervention patients were more likely to receive third-generation cephalosporins than the postintervention patients (55% vs. 28%; *p*=0.006).

**Figure 2:**
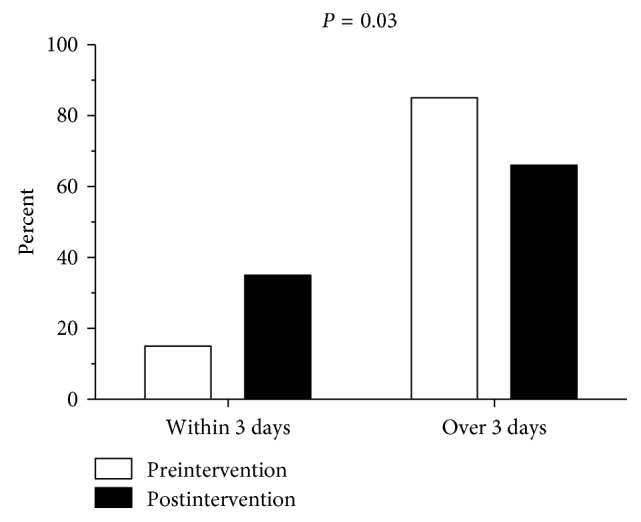
Comparison of the times to antimicrobial susceptibility of the preintervention and postintervention groups.

**Figure 3:**
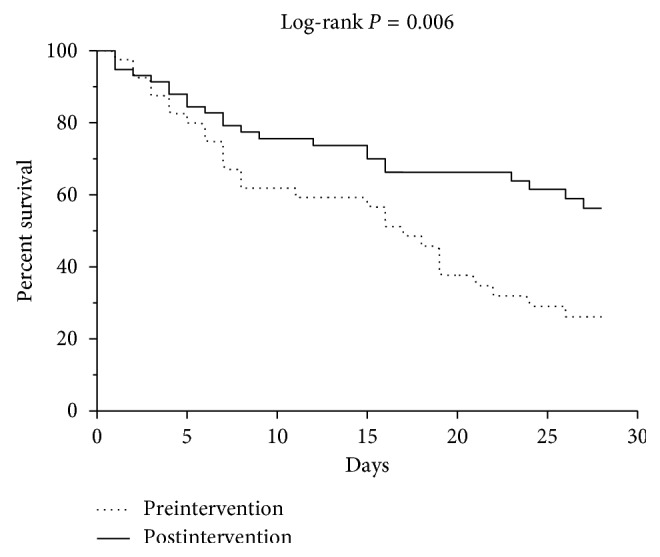
Kaplan–Meier survival curves of the preintervention and postintervention groups.

Table 4 shows the clinical outcomes of the two groups. The postintervention group had a significantly shorter time from blood draw to receipt of the antimicrobial susceptibility results (90.2 ± 32.1 h) than the preintervention group (108.7 ± 43.1 h; *p*=0.02). The postintervention patients were also significantly more likely to receive their antimicrobial susceptibility results within 3 days of blood collection (*p*=0.03) (Figure 2). In addition, before the antimicrobial susceptibility results arrived, the postintervention patients were more likely to be on active antimicrobial therapy (77%) than the preintervention patients (47%; *p*=0.005). Furthermore, the postintervention group had a significantly lower 28-day mortality (23/58, 40%) than the preintervention group (28/40, 70%; *p*=0.003) (Table 4). The preintervention and postintervention groups also differed significantly in terms of Kaplan–Meier survival curves (*p*=0.006) (Figure 3). In both the groups, most deaths were sepsis-related (82% vs. 87%; *p*=0.72) (i.e., septic shock and multiorgan failure were the most common causes of 28-day mortality). The survivors in the two groups did not differ significantly in terms of duration of MV and ICU or hospital stay (Table 4).

Univariate analysis of risk factors that could predict 28-day mortality in the entire cohort showed that the presence of malignancy, vasopressor use, and renal replacement therapy associated significantly with mortality. Conversely, active antimicrobial therapy (odds ratio, 0.38; 95% confidence interval, 0.16–0.95; *p*=0.04) and microorganism identification via MALDI-TOF MS (odds ratio, 0.28; 95% confidence interval, 0.12–0.66; *p*=0.004) were protective factors. While the qSOFA score at ICU admission, use of MV, and Gram-negative bacteremia tended to associate with mortality, these associations did not achieve statistical significance (Table 5).

**Figure 4:**
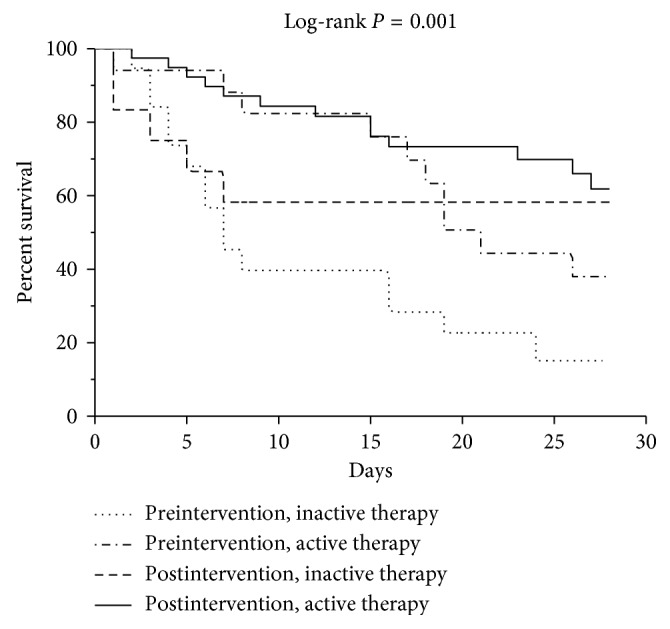
Kaplan–Meier survival curves of patients stratified by pre/postintervention and active/inactive therapy between blood draw and receipt of susceptibility test results.

Figure 4 shows the Kaplan–Meier survival curves of the pre- and postintervention patients after they were stratified according to whether their initial therapy was active or inactive. The preintervention patients who received inactive therapy had the worst prognosis while the postintervention patients who received active therapy had the best prognosis.

## 4. Discussion

The main findings of the present study are as follows. First, when MALDI-TOF MS was implemented for critically ill patients with sepsis, the time between blood draw and receipt of the antimicrobial susceptibility results decreased significantly. Second, MALDI-TOF MS significantly improved the likelihood that the patients were on active antimicrobial therapy by the time the susceptibility report arrived. Third, 28-day mortality dropped significantly in the postintervention group when compared with the preintervention group. To the best of our knowledge, this study expands the findings of previous studies that suggest that MALDI-TOF MS improves the clinical outcomes in patients with bacteremia in general [10, 11, 14, 15]. Moreover, it shows that these benefits of MALDI-TOF MS also apply to critically ill patients with bacteremia.

Identification of the causative organisms is central to the treatment of bloodstream infection. Routine phenotypic identification can involve sample incubation, Gram-staining, subculturing, susceptibility analysis, and analysis of various biochemical reactions [5]. This process can take several days, which is not satisfactory for critical illnesses such as sepsis [19]. The long delays associated with conventional methods may oblige physicians to begin empirical antimicrobial therapy that may not be optimal for the specific infection [6]. This notion is supported by our finding that while 55% of preintervention patients received third-generation cephalosporins as their empirical antibiotic therapy, only 47% were actually on active antimicrobial therapy at the time of susceptibility reporting. MALDI-TOF MS can rapidly identify a very large number of organisms via a relatively simple process, namely, by comparing the mass spectrum produced by the laser ionization of an isolate with spectra held in a reference database. It is accurate: its diagnostic sensitivity is 76–98% and its specificity is over 96% [20]. Consequently, implementation of MALDI-TOF MS decreases the time to organism identification by more than 1 day compared to conventional methods [10, 11]. These findings may help to explain why the postintervention group in our study was much more likely to be receiving active antimicrobial therapy by the time the susceptibility report came in (77% vs. 47%).

Several studies have described the clinical impact of MALDI-TOF MS in bacteremia and/or candidemia [10, 11, 13–15]. In the study conducted by Vlek et al., MALDI-TOF MS decreased the time to identification and improved the rate of appropriate therapy within 24 h of blood culture positivity. However, the authors did not evaluate the impact of MALDI-TOF MS on clinical outcomes. In addition, only 20-21% of their patients were admitted to the ICU [13]. Perez et al. integrated MALDI-TOF MS with antimicrobial stewardship team intervention and showed that these interventions reduced the hospitalization stay and total hospital costs. However, only 16 of their patients (7%) were admitted to the ICU ≥48 h after bacteremia onset [10]. Huang et al. conducted a study with a similar design and showed that MALDI-TOF MS with antimicrobial stewardship team intervention decreased the time to identification, the time to effective and optimal therapy, the mortality, the ICU stay, and the frequency of recurrent bacteremia. However, only 12% (60/501) of their patients had hemodynamic instability that required vasopressors [11]. By contrast, in the present study, all patients were admitted to the ICU, and many of them were treated with MV (56%), vasopressors (69%), and/or renal replacement therapy (39%). Moreover, in nearly half of our patients, pneumonia was the source of the bacteremia. This is known to associate strongly with mortality in patients with sepsis [21]. These findings may help to explain the relatively higher mortality rate of our patients (70% in the preintervention group and 40% in the postintervention group) than of those in previous studies (e.g., 20% and 15% in the respective pre- and postintervention groups in the study by Huang et al. [11]). These findings also suggest that the present study represents rapid patient management in severe infections better than previous studies.

In several studies, an antimicrobial stewardship team was implemented in conjunction with rapid diagnostic testing to enhance antimicrobial management [10, 11, 14, 15]. The antimicrobial stewardship activities included real-time notification of a team member (an infectious diseases physician or pharmacist) when the causative organism was identified (this service was available 24 hours per day, 7 days per week), review of the electronic medical records of the patients, and recommendations to the treating physician regarding the most effective, targeted-antimicrobial therapy. However, our hospital does not have the 24 hours per day and 7 days per week notification service; it also lacks an antimicrobial stewardship team. This may explain, at least in part, why we tended to have longer times to susceptibility reporting (108 and 90 h in the pre- and postintervention groups, respectively) than other studies (e.g., 48 and 23 h, respectively, in the study by Lockwood et al. [15]). Thus, although our finding that rapid diagnostics (even without an antimicrobial stewardship team) improved outcomes is encouraging, these observations make clear that administrative and/or financial support and expertise (e.g., in the form of dedicated infectious disease specialists) are all required to maximize the efficiency of MALDI-TOF MS [22, 23].

The present study has several limitations. First, its single-center nonrandomized design increases the risk of selection bias. Indeed, there were differences between the intervention and control groups in terms of baseline characteristics that could have influenced the clinical outcomes. For instance, malignancies were more frequently observed in the preintervention group. However, most deaths in our study were sepsis-related and not related to the underlying disease of the patients. Thus, this limitation does not undermine the main conclusion of the study. Second, the sample size was relatively small. Power calculation shows that to confirm the 18 h shorter time to susceptibility reporting with 80% power, 5% error rate, and 108 h baseline control time (same as our study), it would take a total of 142 patients (71 in each group) to complete the study. Third, we were unable to record the exact time of organism identification by MALDI-TOF MS because the result time was not captured in our electronic medical record or microbiology system. Thus, the time to organism identification (which would be expected to be much shorter in the postintervention group than in the preintervention group) could not be analyzed. Fourth, while qSOFA criteria are clinically valuable, they can be imperfect markers of sepsis. For instance, a patient can have a qSOFA ≥2 in acute conditions other than sepsis (e.g., hypovolemia, severe heart failure, or large pulmonary embolism) [24]. A recent study showed that to improve sepsis identification, the qSOFA score should be used in combination with laboratory tests such as procalcitonin [25]. However, measurements of procalcitonin were not routine clinical practice in our institution, especially in the preintervention period. Nevertheless, such bias was minimized in our study by the fact that we only included patients with a documented bloodstream infection.

## 5. Conclusions

Our results show that implementation of MALDI-TOF MS accelerated susceptibility testing, probably by expediting microorganism identification, and that this improved the frequency of active antimicrobial therapy and reduced mortality in patients with sepsis. However, study limitations (and the relatively high cost and complexity of MALDI-TOF MS compared to conventional methods) suggest that further studies are required to justify the routine use of MALDI-TOF MS for patients with sepsis.

## Figures and Tables

**Table 1:** Baseline characteristics of the preintervention and postintervention groups.

Variable	Preintervention group (*n*=40)	Postintervention group (*n*=58)	*p*
Age (years)	63.2 ± 14.0	66.4 ± 13.6	0.26
Male (sex)	25 (63)	30 (52)	0.29
Comorbidity			
Diabetes	8 (20)	21 (36)	0.08
Chronic heart failure	3 (8)	10 (17)	0.16
Cerebrovascular accident	2 (5)	7 (12)	0.30
Chronic pulmonary disease	2 (5)	6 (10)	0.47
Liver cirrhosis	3 (8)	6 (10)	0.73
Chronic kidney disease	3 (8)	2 (3)	0.40
Malignancy	18 (45)	9 (16)	0.001
No. of SIRS criteria met at ICU admission	3.3 ± 0.7	3.4 ± 0.7	0.59
qSOFA score at ICU admission	2.3 ± 0.5	2.3 ± 0.5	0.98
Altered mentation	28 (70)	44 (76)	0.52
Respiratory rate ≥22 breaths/min	32 (80)	49 (85)	0.57
Systolic blood pressure ≤100 mmHg	33 (83)	42 (72)	0.25
Within 72 h of ICU admission			
Mechanical ventilation	24 (60)	31 (53)	0.52
Neuromuscular blockers	6 (15)	14 (24)	0.27
Vasopressor use	28 (70)	40 (69)	0.91
Renal replacement therapy	20 (50)	18 (31)	0.06
Source of bacteremia			
Respiratory	17 (43)	25 (43)	0.95
Intra-abdominal	11 (28)	15 (26)	0.86
Genitourinary	4 (10)	5 (9)	>0.99
Skin and soft tissue/bone and joint	3 (8)	10 (17)	0.16
Vascular catheter	2 (5)	4 (7)	>0.99

The data are presented as mean ± standard deviation or number (percentage) of patients. *p* values indicate the results of comparing the preintervention and postintervention groups by using Student's *t*-, chi-squared, or Fisher's exact tests. SIRS: systemic inflammatory response syndrome; qSOFA: quick Sequential Organ Failure Assessment; ICU: intensive care unit.

**Table 2:** Microorganisms identified.

Variable	Preintervention group (*n*=40)	Postintervention group (*n*=58)	*p*
Gram-positive organisms	16 (40)	33 (57)	0.10
Gram-negative organisms	21 (53)	24 (41)	0.28
Yeast	6 (15)	4 (7)	0.31
Polymicrobial	6 (15)	4 (7)	0.31
Multidrug-resistant organisms			
MRSA	5 (13)	2 (3)	0.12
VRE	2 (5)	0	0.16
ESBL	6 (15)	6 (10)	0.54
CRPA	1 (3)	1 (2)	>0.99
CRAB	1 (3)	0	0.41

The data are presented as number (percentage) of patients. *p* values indicate the results of comparing the preintervention and postintervention groups by using chi-squared or Fisher's exact tests. MRSA: methicillin-resistant *Staphylococcus aureus*; VRE: vancomycin-resistant enterococci; ESBL: extended-spectrum beta-lactamase-producing organism; CRPA: carbapenem-resistant *Pseudomonas aeruginosa*; CRAB: carbapenem-resistant *Acinetobacter baumannii*.

**Table 3:** Antimicrobials used from the time of blood draw to susceptibility reporting.

Variable	Preintervention group (*n*=40)	Postintervention group (*n*=58)	*p*
Glycopeptide	15 (38)	30 (52)	0.17
Carbapenem	16 (40)	28 (48)	0.42
Third-generation cephalosporin	22 (55)	16 (28)	0.006
Antipseudomonal penicillin	8 (20)	18 (31)	0.22
Quinolone	9 (23)	14 (24)	0.85
Fourth-generation cephalosporin	4 (10)	5 (9)	>0.99
Linezolid	1 (3)	4 (7)	0.65
Aminoglycoside	2 (5)	2 (3)	>0.99
Colistin	2 (5)	0	0.16
Tigecycline	0	2 (3)	0.51

The data are presented as number (percentage) of patients. *p* values indicate the results of comparing the preintervention and postintervention groups by using chi-squared or Fisher's exact tests.

**Table 4:** Clinical outcomes of the preintervention and postintervention groups.

Variable	Preintervention group (*n*=40)	Postintervention group (*n*=58)	*p*
Time from blood draw to susceptibility reporting (h)	108.7 ± 43.1	90.2 ± 32.1	0.02
Active antimicrobial therapy at susceptibility reporting (*n*=36 or 51)^a^	17 (47)	39 (77)	0.005
28-day mortality after ICU admission	28 (70)	23 (40)	0.003
Cause of death (*n*=28 or 23)			0.72
Sepsis-related	23 (82)	20 (87)	—
Other causes	5 (18)	3 (13)	—
In survivors (*n*=12 or 35)			
MV duration (d) (*n*=6 or 19)	16.8 ± 17.5	17.8 ± 27.5	0.93
ICU length of stay (d)	17.1 ± 13.5	22.1 ± 20.5	0.44
Hospital length of stay (d)	49.5 ± 35.0	52.5 ± 41.1	0.82

The data are presented as mean ± standard deviation or number (percentage) of patients. *p* values indicate the results of comparing the preintervention and postintervention groups by using Student's *t*-, chi-squared, or Fisher's exact tests. ICU: intensive care unit; MV: mechanical ventilation. ^a^Patients with incomplete data regarding the microorganisms or antibiotics were excluded.

**Table 5:** Univariate analysis of factors predicting 28-day mortality.

Variable	Unadjusted OR (95% CI)	*p*
Malignancy	8.83 (2.76–28.27)	<0.001
qSOFA score at ICU admission	1.88 (0.79–4.46)	0.15
Use of mechanical ventilation	2.08 (0.93–4.69)	0.08
Vasopressor use	3.77 (1.50–9.47)	0.005
Use of renal replacement therapy	5.57 (2.23–13.88)	<0.001
Gram-negative organisms	2.15 (0.95–4.84)	0.07
Active antimicrobial therapy	0.38 (0.16–0.95)	0.04
Identification via MALDI-TOF MS	0.28 (0.12–0.66)	0.004

OR: odds ratio; CI: confidence interval; qSOFA: quick Sequential Organ Failure Assessment; ICU: intensive care unit; MALDI-TOF: matrix-assisted laser desorption/ionization time-of-flight mass spectrometry.

## Data Availability

The data used to support the findings of this study are restricted by the Institutional Review Board of Pusan National University Hospital to protect the privacy of the patients. Data are available from the corresponding author for researchers who meet the criteria for access to confidential data.
